# Associations of psychological resilience and change fatigue with innovative behavior among hemodialysis nurses in Chinese public hospitals: a cross-sectional mediation analysis

**DOI:** 10.3389/fpsyg.2026.1756479

**Published:** 2026-09-23

**Authors:** Wei Qing, Jiquan Zhang, Haiyan Yang, Zhenfan Liu, Zhaohua Zou

**Affiliations:** 1 Nephrology Department, Deyang People’s Hospital, Deyang, Sichuan, China; 2 Department of Respiratory Medicine, Affiliated Hospital of Southwest Medical University, Luzhou, Sichuan, China; 3 Nursing Department, Deyang People's Hospital, Deyang, Sichuan, China

**Keywords:** change fatigue, hemodialysis nurses, innovative behavior, mediation analysis, psychological resilience

## Abstract

**Background:**

Against the backdrop of continuously rising demand for healthcare services and an increasingly scarce nursing workforce, innovation and development within the nursing profession have become particularly urgent. Enhancing nurses’ capacity for innovative practice is not only crucial for optimizing nursing efficiency but also directly impacts patient safety and the sustainable development of hospitals. This represents a critical issue that requires urgent attention in China’s healthcare management sector.

**Objective:**

This study examined the associations of psychological resilience and change fatigue with innovative behavior among hemodialysis nurses in Chinese public hospitals, and explored whether change fatigue was statistically indirectly associated with the relationship between psychological resilience and innovative behavior, aiming to provide evidence for developing strategies to enhance nurses’ innovative practices.

**Methods:**

This cross-sectional study recruited 338 hemodialysis nurses from 14 public hospitals in Sichuan Province, China, between March and June 2024. Data collection employed a demographic questionnaire, the Simplified Psychological Resilience Scale, the Change Fatigue Scale, and the Nurse Innovation Behavior Scale. An exploratory cross-sectional mediation analysis was performed using the SPSS PROCESS macro developed by Hayes.

**Results:**

Innovative behaviors among hemodialysis nurses showed a positive correlation with total psychological resilience scores (*r* = 0.558, *p* < 0.001) and a negative correlation with total change fatigue scores (*r* = −0.383, *p* < 0.001). Psychological resilience itself exhibited a negative correlation with change fatigue (*r* = −0.467, *p* < 0.001). Within the specified statistical model, the unstandardized indirect association between psychological resilience and innovative behavior through change fatigue was 0.113, and this indirect association accounted for 24.4% of the total association.

**Conclusion:**

Psychological resilience was not only associated with the innovative behavior of hemodialysis nurses in public hospitals, but was also statistically indirectly associated with this behavior through change fatigue. Measures should be implemented at both the individual and managerial levels to enhance nurses’ psychological resilience and reduce their change fatigue, which may be linked to strengthened proactive innovative behavior and the high-quality development of nursing services.

## 1 Introduction

Nurses serve as an indispensable driving force in the evolution of medical reform and development, with their level of innovation directly impacting a hospital’s overall capacity for innovation (Xiang et al., 2024). As global population aging intensifies and chronic disease patients surge, public health demands are rapidly increasing. However, according to the latest data from the National Bureau of Statistics of China, the number of registered nurses in China stood at only 5.63 million by the end of 2023 (Ren, 2024). The imbalance in the nurse-to-patient ratio has exacerbated the contradiction between supply and demand in healthcare services. Against this backdrop, innovation and development within the nursing profession have become particularly urgent. The National Nursing Development Plan (2021–2025) issued by the National Health Commission explicitly states that to adapt to the new landscape of nursing development, it is essential to grasp the characteristics of nursing work, improve the nursing service system, innovate nursing service models, advance nursing service reforms, and sustain the development of nursing services (Commission NH, 2022). The convergence of cutting-edge technologies such as artificial intelligence, mobile health technology, and big data has revitalized the nursing industry while presenting new challenges. The boundaries of nursing services are being redefined, expanding from traditional clinical care to encompass chronic disease management, long-term care, palliative care, health counseling, and even public health education. Innovation in nursing management, technology, and services has become an essential choice for advancing clinical practice and a key approach to alleviating healthcare pressure and enhancing nursing efficiency.

Nursing innovation refers to the process by which nurses generate new ideas, develop novel methods, technologies, and services, and subsequently introduce and apply these innovations in their practice after gaining support from others (Asurakkody and Shin, 2018). As the healthcare professionals who interact most frequently and intimately with patients, nurses’ innovative behaviors constitute an essential capability for the dynamic development of nursing staff within healthcare systems. Their innovative actions play an irreplaceable and vital role. Research indicates that nursing innovation enhances patient care quality, reduces healthcare costs, and improves patient outcomes (Guo et al., 2025). However, the level of nursing innovation is generally low. Yan’s survey (Yan et al., 2020) of 4,677 nurses from 18 top-tier hospitals in China revealed an average innovation score of (3.06 ± 0.66). Results indicate nurses face challenges in obtaining support and realizing ideas during the innovation process, making it difficult to complete the full innovation cycle. International comparative studies reveal that the average innovation score for registered nurses in the United States was 3.17 (Dy et al., 2016), while Danish intensive care unit nurses scored (3.21 ± 0.74) (Li-Ying et al., 2016). This indicates that both domestic and international nurses have room for improvement in their innovation capabilities and levels, necessitating further enhancement. Therefore, systematically exploring and implementing scientific, effective incentive strategies to comprehensively elevate nurses’ innovative behavior has become an urgent priority in China’s healthcare management field.

The American Psychological Association defines psychological resilience as an individual’s capacity to maintain positive emotions and effective coping influencing path in adverse situations such as adversity, stress, or significant challenges, ultimately achieving positive psychological outcomes (Connor and Davidson, 2003). As a key positive psychological trait, psychological resilience enables individuals to flexibly mobilize internal and external resources, rapidly adjust cognitive and behavioral strategies, thereby enhancing problem-solving probabilities and providing psychological momentum for innovative behavior. Empirical research by Liu (2019) on nursing populations further revealed a significant positive correlation between psychological resilience levels and innovative behavior. It suggested that nursing managers could enhance nurses’ psychological resilience through intervention strategies to promote their innovative behavior. However, this study did not fully elucidate the underlying influencing path through which psychological resilience influences innovative behavior, and its mediating pathways remain to be explored in depth.

Change fatigue refers to the phenomenon where rapid, continuous changes in the workplace cause emotional exhaustion, stress, and burnout among employees or managers, thereby diminishing their work engagement and behavioral performance (Lv et al., 2025). According to Conservation of Resources Theory (COR) (Hobfoll, 1989), frequent organizational changes continuously deplete individuals’ cognitive, emotional, and material resources. When resource expenditure consistently exceeds returns over time, individuals enter a state of resource depletion, exhibiting high levels of psychological fatigue and diminished motivation, ultimately inhibiting innovative behavior (Zhang et al., 2025). Psychological resilience, as an individual’s positive psychological capital, enhances emotional regulation and stress reframing abilities (Li and Quan, 2025). Brown et al. (2018) found a significant negative correlation between nurses’ psychological resilience and change fatigue. Highly resilient individuals demonstrate greater adaptability and support for change in transformative contexts, thereby effectively mitigating change fatigue. According to the Stimulus-Organism-Response (SOR) theory (Mehrabian and Russell, 1974), when confronted with change stimuli, individuals with high psychological resilience can employ positive cognitive and emotional regulation strategies to mitigate negative effects arising from stress and fatigue. This activates positive coping pathways, thereby facilitating innovative behavior. Therefore, change fatigue may mediate the relationship between psychological resilience and innovative behavior—that is, psychological resilience indirectly enhances nurses’ innovative behavior by reducing change fatigue.

Research on innovative behavior predominantly focuses on corporate employees, with relatively few studies examining nurses’ innovative practices. Domestic researchers emphasize that fostering individual innovation awareness and spirit among nursing staff, alongside supporting innovative nursing theories and techniques, serves as a driving force for the discipline’s advancement. To date, no comprehensive study has explored the relationship among psychological resilience, change fatigue, and innovative behavior among hemodialysis nurses. This study integrates COR and SOR to explain the psychological association of innovative behavior of hemodialysis nurses, expanding the application of these two theories in the field of nursing management. Previous studies have mostly focused on nurses in general wards or employees in enterprises. Hemodialysis nurses face triple pressures of updated dialysis technology, frequent policy adjustments, and high workloads. The influencing factors of their innovative behavior differ from those of nurses in general wards. The research hypotheses are as follows: H1: Psychological resilience is positively associated with innovative behavior; H2: Psychological resilience is negatively associated with change fatigue; H3: Change fatigue is statistically indirectly associated with the relationship between psychological resilience and innovative behavior. The Hypothesis model is shown in Figure 1.

**Figure 1:**
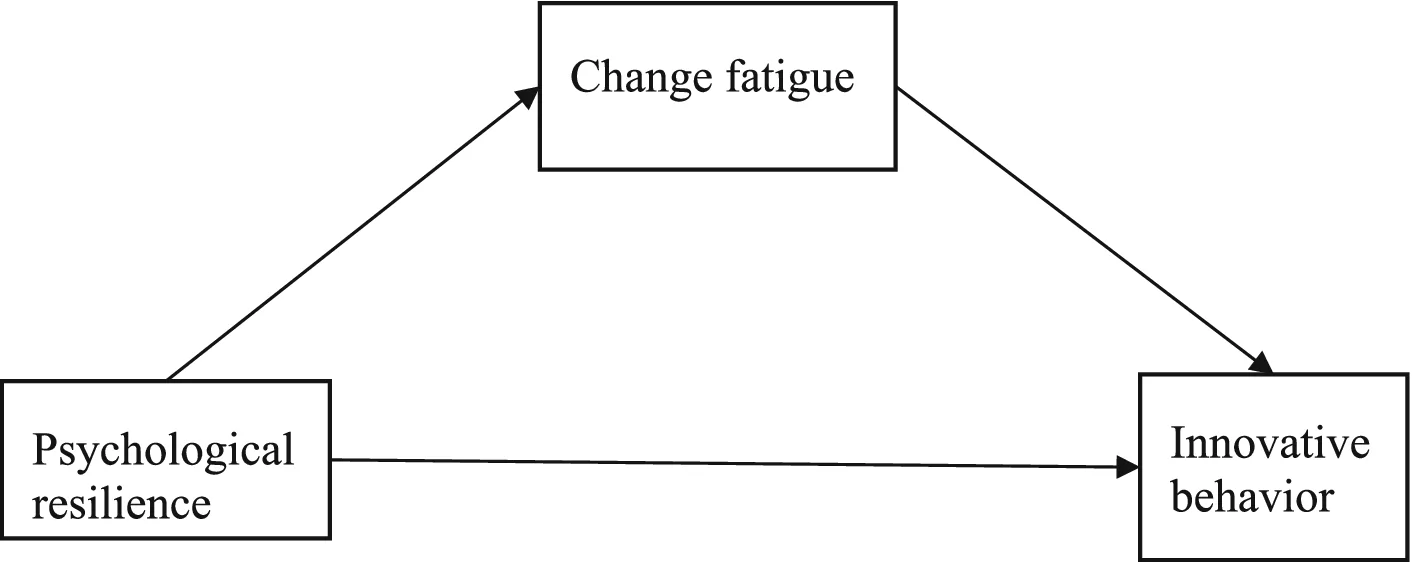
Hypothesis model.

## 2 Methods

### 2.1 Participants

This study employed convenience sampling to conduct a questionnaire survey among 338 hemodialysis nurses working in 14 public hospitals in Sichuan Province, China, from March to June 2024. Inclusion criteria: ① Age ≥ 18 years; ② Possession of a valid nursing license with ≥ 1 year of hemodialysis nursing experience; ③ Informed consent and willingness to participate. Exclusion criteria: Nurses on leave or in training. The required sample size was calculated using the formula *n* = [u_α_*σ/*δ*]^2^, with *α* = 0.05, *u_α_* = 1.96, allowable error *δ* = 0.8, and *σ* = 6.86 based on prior research (Li et al., 2023). The calculated sample size was 283 participants. Accounting for a 10% anticipated non-response or invalid-response rate, the target sample size was set at 312 participants. In total, 345 questionnaires were distributed; 7 were excluded as invalid; and 338 valid questionnaires were analyzed.

### 2.2 Measures

#### 2.2.1 General information questionnaire

Developed independently by research team members following discussions on study objectives, it primarily covers general demographic information and job characteristics. General demographic information includes: gender, age, marital status, and Educational level. Job characteristics include: Hospital level, Personal monthly income, employment method, department atmosphere (it was assessed with a single study-specific, self-rated item with three categories (disharmonious/general/harmonious), not a validated scale), scientific research project and duration of professional training.

#### 2.2.2 Simplified Psychological Resilience Scale

The 25-item Psychological Resilience Scale developed by Connor et al. in 2003 was condensed into a simplified version by Campbells-Still et al., who selected 10 items. Chinese scholars Wang et al. (2010) translated and revised this into the Chinese Simplified Psychological Resilience Scale. The scale employs a 5-point Likert scoring method, with responses ranging from “Never” to “Always” scored from 1 to 5 points, respectively. All items are scored positively. The sum of the 10 items yields the total psychological resilience score, ranging from 10 to 50 points. Higher scores indicate greater psychological resilience. The Cronbach’s *α* coefficient for this scale is 0.91 (Li et al., 2015). In this study, the Cronbach’s alpha was 0.978, and McDonald’s omega was 0.977.

#### 2.2.3 Change Fatigue Scale (CFS)

Developed by Bernerth et al. (2011), this 6-item scale employs a 7-point Likert scale. Responses ranging from “Strongly Disagree” to “Strongly Agree” are scored from 1 to 7 points, respectively. Higher scores indicate greater employee fatigue toward organizational change. The scale’s Cronbach’s alpha coefficient is 0.940. In this study, the Cronbach’s alpha for this scale was 0.895, and McDonald’s omega was 0.910.

#### 2.2.4 Nurse Innovation Behavior Scale

Developed by Bao et al. (2012), this 10-item scale comprises three dimensions: idea generation, support acquisition, and idea implementation. The scale consists of 10 items rated on a 5-point Likert scale; the total score from 10 to 50, with higher scores indicating more prominent innovative behavior. The scale’s Cronbach’s *α* coefficient is 0.906. In this study, the Cronbach’s alpha for this scale was 0.951, and McDonald’s omega was 0.952.

### 2.3 Data collection methods

First, the research team members drafted the questionnaire content, including the title, introduction, survey objectives, significance, methodology, and instructions for completion. Based on preliminary survey results, revisions were made to generate the final official version of the questionnaire. Next, the project leader contacted department heads at relevant hospitals, explaining the purpose, content, and significance of the survey. With their consent, these individuals were trained as survey administrators. Finally, participants completed the questionnaire voluntarily and with informed consent, tailored to their individual circumstances. In total, 345 questionnaires were distributed; 7 were excluded as invalid; and 338 valid questionnaires were analyzed, a valid response rate of 98.0%.

### 2.4 Statistical methods

The analysis was conducted using SPSS 25.0 statistical software. In this study, the results of assessing multicollinearity through variance inflation factor (VIF) and tolerance were presented. All VIF values were less than 2.0, and tolerance values were all greater than 0.5, indicating that there were no serious multicollinearity issues. The normality of residuals was evaluated through Shapiro–Wilk test and visual inspection of Q-Q plots; linearity was assessed through scatter plots and partial residual plots; homoscedasticity was evaluated through Breusch-Pagan test and residual-fitted value plots. Descriptive analysis, non-parametric tests, and multivariate linear hierarchical regression analysis were used to explore the relationships between variables. The mediation effect analysis was conducted using the SPSS macro program PROCESS developed by Hayes. The PROCESS Model 4 analysis has adjusted for factors such as gender, hospital level, personal monthly income, employment method, department atmosphere, scientific research project, and duration of professional training. *p* < 0.05 was considered statistically significant.

## 3 Results

### 3.1 General demographic characteristics

A total of 338 nurses were surveyed. Gender: 27 males (8.0%), 311 females (92.0%); Age: Ranged from 22 to 54 years old, predominantly aged 30–39 (54.4%); Educational level: Undergraduate (80.8%); Marital status: married (79.3%); Personal monthly income: 5,000–7,999 (49.1%); Employment method: Contract employment (85.2%); only 9 individuals (2.7%) had two or more research projects. Professional learning time was primarily 1–2 h (49.7%).

### 3.2 Scores for psychological resilience, change fatigue, and innovative behavior among hemodialysis nurses

The scores for psychological resilience, change fatigue, and innovative behavior among 338 hemodialysis nurses are detailed in Table 1.

**Table 1:** Scores for psychological resilience, change fatigue, and innovative behavior among hemodialysis nurses (*n* = 338).

Variable	Number of entries	Score range	Score
Psychological resilience	10	10 ~ 50	40.00 (31.00, 43.00)
Change fatigue	6	6 ~ 42	24.00 (19.00, 27.00)
Innovative behavior	10	10 ~ 50	32.00 (30.00, 39.00)
Idea generation	3	3 ~ 15	11.00 (9.00, 12.00)
Support acquisition	4	4 ~ 20	12.00 (12.00, 15.00)
Idea implementation	3	3 ~ 15	9.00 (9.00, 12.00)

### 3.3 Comparison of innovative behavior scores among hemodialysis nurses with different characteristics

Univariate analysis revealed statistically significant differences in the total innovative behavior scores of hemodialysis nurses across various characteristics, including gender, hospital level, Personal monthly income, employment method, departmental atmosphere, scientific research project, and duration of specialized training (all *p* < 0.05). Details are provided in Table 2.

**Table 2:** Comparison of innovation behavior scores among hemodialysis nurses with different characteristics.

Variable	Item	*N* (%)	Score M (P25, P75)	*Z*	*p*
Gender	Male	27 (8.0)	39.00 (30.00, 44.00)	−2.270	0.023
	Female	311 (92.0)	32.00 (30.00, 38.00)		
Hospital level	Grade III Class A	246 (72.8)	34.00 (30.00, 40.00)	21.405	<0.001
	Grade III Class B	43 (12.7)	31.00 (30.00, 36.00)		
	Class A and below	49 (14.5)	30.00 (29.50, 32.00)		
Age (years)	20–29	96 (28.4)	31.00 (30.00, 39.00)	2.085	0.555
	30–39	184 (54.4)	32.50 (30.00, 39.00)		
	40–49	45 (13.3)	32.00 (30.00, 39.00)		
	≥50	13 (3.8)	34.00 (32.50, 39.00)		
Educational level	Junior college	61 (18.0)	32.00 (30.00, 36.00)	3.991	0.136
	Undergraduate	273 (80.8)	32.00 (30.00, 39.00)		
	Postgraduate degree or above	4 (1.2)	38.50 (35.50, 47.50)		
Marital status	unmarried	57 (16.9)	31.00 (30.00, 35.50)	3.953	0.139
	Married	268 (79.3)	33.00 (30.00, 40.00)		
	Divorce	13 (3.8)	30.00 (29.00, 37.00)		
Personal monthly income	<5,000	97 (28.7)	30.00 (29.00, 33.00)	31.967	<0.001
	5,000 ~ 7,999	166 (49.1)	34.00 (30.00, 40.00)		
	8,000 ~ 9,999	61 (18.0)	37.00 (31.00, 40.00)		
	≥10,000	14 (4.1)	34.50 (29.50, 39.25)		
Employment method	Formal establishment	50 (14.8)	37.00 (32.00, 40.00)	−3.174	0.001
	Contract employment	288 (85.2)	31.50 (30.00, 38.00)		
Department atmosphere	Disharmony	7 (2.1)	30.00 (27.00, 30.00)	32.884	<0.001
	General	74 (21.9)	30.00 (30.00, 32.00)		
	Harmony	257 (76.0)	34.00 (30.00, 40.00)		
Scientific research project	Zero	244 (72.2)	31.00 (30.00, 37.00)	15.736	<0.001
	One	85 (25.1)	35.00 (31.00, 40.00)		
	Two or more	9 (2.7)	32.00 (30.00, 50.00)		
Duration of professional training (hour/day)	<1 h	147 (43.5)	31.00 (30.00, 35.00)	19.221	<0.001
	1 ~ 2 h	168 (49.7)	34.00 (30.00, 40.00)		
	2 ~ 3 h	14 (4.1)	34.00 (30.00, 38.25)		
	≥3 h	9 (2.7)	40.00 (31.50, 48.00)		

### 3.4 Correlation analysis of psychological resilience, change fatigue, and innovative behavior among hemodialysis nurses

Spearman correlation analysis revealed that innovative behavior among hemodialysis nurses positively correlated with total psychological resilience scores (*r =* 0.558, *p* < 0.001) and negatively correlated with total change fatigue scores (*r =* −0.383, *p* < 0.001). Psychological resilience itself negatively correlated with change fatigue (*r =* −0.467, *p* < 0.001). Details are provided in Table 3.

**Table 3:** Correlation analysis of psychological resilience, change fatigue, and innovative behavior among hemodialysis nurses.

Variable	M(P25, P75)	Change fatigue	Psychological resilience	Innovative behavior
Change fatigue	24.00 (19.00, 27.00)	1		
Psychological resilience	40.00 (31.00, 43.00)	−0.467 **	1	
Innovative behavior	32.00 (30.00, 39.00)	−0.383 **	0.558**	1

**p < 0.001.

### 3.5 The mediation analysis of change fatigue in the relationship between psychological resilience and innovative behavior among hemodialysis nurses

In this study, the common latent factor (CLF) method was employed to assess the potential common method bias. The analysis was conducted using maximum likelihood estimation. A baseline three-factor confirmatory factor analysis was first specified, in which the 26 items (10 resilience, 6 change fatigue, and 10 innovative behavior items) loaded on their respective substantive factors, with the three factors allowed to correlate. A latent common method factor was then added, on which all 26 items loaded; following the method factor was specified as uncorrelated with the substantive factors, and the model was identified by fixing the method factor variance to 1 and constraining the sum of the method factor loadings to zero. The fit of the method model was compared with that of the baseline model. Common method bias is suggested when the method factor accounts for a substantial proportion of variance (> 25%), when method loadings are large, and when model fit improves substantially (ΔCFI ≥ 0.05). The results showed that the proportion of variance explained by the method factor alone was 4.14%, the root mean square (RMS) of the method factor loadings was 0.196. Additionally, after incorporating the common method factor, the model fit improved minimally (ΔCFI < 0.01, ΔTLI < 0.01), and the average correlation coefficient among the three scales was 0.469, much lower than the 0.80 benchmark indicative of severe common method bias. These results indicate that common method bias is unlikely to be a serious concern in this study. Nevertheless, because all variables were collected simultaneously through self-report measures from a single source, common method bias cannot be completely excluded. To address the issue of potential non-independence of the observed values, we conducted a sensitivity analysis based on the design effect (DE = 1 + (m̄ − 1) × ICC), where m̄ represents the average clustering size (338/14 ≈ 24.1). When the within-group correlation coefficient (ICC) was assumed to be 0.01, 0.05, and 0.10 respectively, the corresponding design effects were 1.23, 2.16, and 3.31. After amplifying the standard error of the unstandardized indirect association (BootSE = 0.028) by the square root of these design effects, the approximate 95% confidence intervals were [0.052, 0.174], [0.033, 0.193], and [0.013, 0.213] respectively. Even in the most conservative scenario (ICC = 0.10), the indirect association between psychological resilience and innovative behavior through fatigue remained statistically significant (the confidence interval did not include 0). These results suggest that hospital clustering is unlikely to have a substantial impact on our research findings.

The mediation analysis was conducted using the Hayes PROCESS macro (Model 4), using the percentile Bootstrap method with bias correction, 5,000 replications were conducted, and the 95% confidence interval was calculated. The analysis adjusted for gender, hospital level, personal monthly income, employment method, department atmosphere, scientific research project, and duration of professional training. The results showed that psychological resilience was negatively correlated with change fatigue (path a: *B* = −0.395, SE = 0.048, 95% CI [−0.489, −0.300], *p* < 0.001); change fatigue was negatively correlated with innovative behavior (path b: *B* = −0.286, SE = 0.051, 95% CI [−0.387, −0.185], *p* < 0.001); after adding change fatigue, the direct effect of psychological resilience on innovative behavior remained significant (path c’: *B* = 0.350, SE = 0.049, 95% CI [0.253, 0.446], *p* < 0.001). The total effect of psychological resilience on innovative behavior was also significant (path c: *B* = 0.463, SE = 0.047, 95% CI [0.371, 0.555], *p* < 0.001). The indirect effect of psychological resilience on innovative behavior through change fatigue was significant (*B* = 0.113, BootSE = 0.028, 95% CI [0.062, 0.171]), indicating a statistically significant indirect association, with the indirect effect accounting for 24.4%, as shown in Tables 4, 5 and Figure 2.

**Table 4:** Regression model for the mediation analysis of change fatigue in the relationship between psychological resilience and innovative behavior among hemodialysis nurses.

Regression equation (*N* = 338)		Fitting index			Unstandardized coefficient			Standardized coefficient		
Outcome variable	Predictor variable	*R*	*R* ^2^	F(df)	*B*	SE	95% CI	*β*	95% CI	*t*
Innovative behavior	Psychological resilience	0.614	0.377	*F*(8329) = 24.922	0.463	0.047	[0.371, 0.555]	0.493	[0.395, 0.591]	9.895**
Change fatigue	Psychological resilience	0.543	0.295	F(8329) 17.200	−0.395	0.048	[−0.489, −0.300]	−0.436	[−0.540, −0.332]	−8.223**
Innovative behavior	Psychological resilience	0.657	0.431	*F* (9328) = 27.620	0.350	0.049	[0.253, 0.446]	0.373	[0.270, 0.476]	7.115**
	Change fatigue	--	--	--	−0.286	0.051	[−0.387, −0.185]	−0.276	[−0.373, −0.179]	−5.569**

**p < 0.001. All models adjusted for gender, hospital level, personal monthly income, employment method, department atmosphere, scientific research project, and Duration of professional training. *B*, unstandardized coefficient; SE, standard error; CI, confidence interval; *β*, standardized coefficient. --, not applicable. df, degrees of freedom.

**Table 5:** Indirect statistical association of change fatigue between psychological resilience and innovative behavior: an exploratory cross-sectional mediation analysis.

Path	Standardized path effects	Unstandardized path effects	Proportion of the total association represented by the indirect association (%)	95%CI	
				Lower limit	Upper limit
Indirect effect	0.120	0.113	24.4%	0.062	0.171
Direct effect	0.373	0.350	75.6%	0.253	0.446
Total effect	0.493	0.463	100.00	0.371	0.555

**Figure 2:**
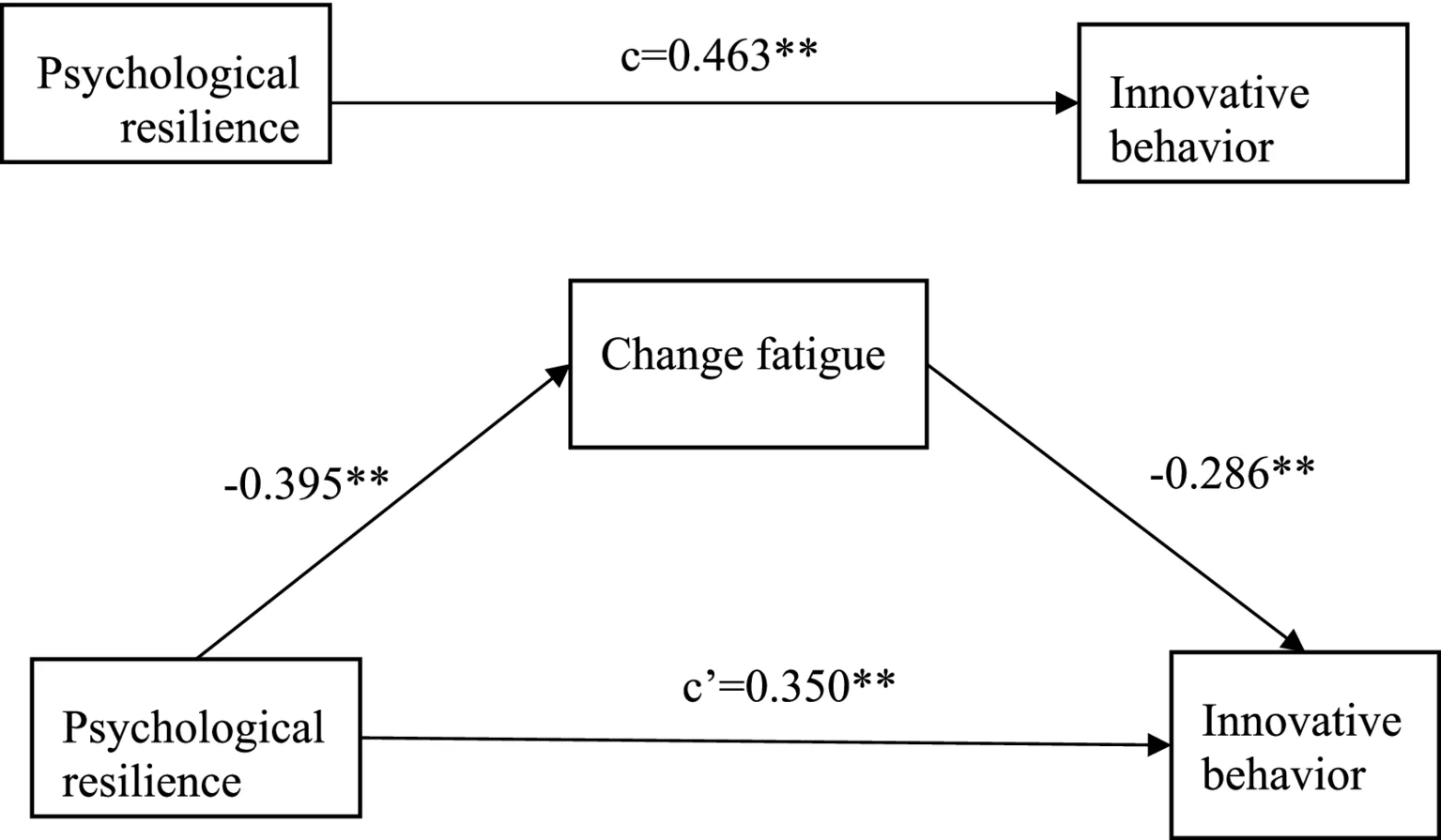
Mediation model.

## 4 Discussion

### 4.1 Current status of psychological resilience, change fatigue, and innovative behavior among hemodialysis nurses

The study findings indicate that hemodialysis nurses scored 40.00 (31.00, 43.00) on the overall psychological resilience scale, with item-level averages exceeding those reported by Li et al. (2024) for this nursing population (2.41 ± 0.58). This discrepancy may stem from the higher proportion of nurses from tertiary hospitals in this study. Tertiary hospitals possess more abundant human and material resources, offer relatively better compensation for nurses, and may provide greater opportunities for international exchanges and professional development. These factors foster stronger professional identity and satisfaction among nurses, enhancing their confidence and coping abilities when facing emergencies, thereby contributing to higher levels of psychological resilience (Gao et al., 2012). Psychological resilience is influenced by multiple factors, including demographics, individual characteristics, and organizational elements. Managers can enhance nurses’ psychological resilience by providing robust organizational support (Cline, 2015), encouraging educational advancement (Campbell-Sills et al., 2009), improving job satisfaction (Thomas et al., 2012), fostering a sense of care and support, alleviating work-related stress, and promoting self-development.

The results of this study indicate that the total change fatigue score for hemodialysis nurses was 24.00 (19.00, 27.00), suggesting moderate levels of change fatigue. This score was higher than the change fatigue score (19.01 ± 3.63) reported by Yu et al. (2025) for nurses at a tertiary public hospital in China. This indicates that the hemodialysis nursing group experiences relatively high levels of change fatigue. Hemodialysis nurses must constantly respond to frequent dialysis machine alarms, acute complications, and occupational exposures, maintaining sustained high mental tension. This undermines their ability to adapt to frequent policy, technical, and procedural changes, making them more susceptible to change fatigue (Cao and Chen, 2021; Pehlivan and Aydın, 2024). Nurses’ change fatigue is influenced by factors such as educational level, psychological factors, and hospital level. Managers can provide targeted care and support to alleviate fatigue levels (Mehrabian and Russell, 1974; Beaulieu et al., 2023).

The results of this study indicate that the total score for innovative behavior among hemodialysis nurses was 32.00 (30.00, 39.00), with the average score for each item lower than that reported by Zhou et al. (2023) for general ward nurses (34 points). Innovative behavior requires further enhancement, and this discrepancy may be related to the severe shortage of hemodialysis nursing staff in China. However, in practice, each nurse is responsible for six or more patients. This forces nurses to focus their primary energy on routine nursing tasks, neglecting the development of their own innovative behaviors. Managers can reasonably allocate nursing staff to reduce nurses’ workload, conduct relevant research training, and provide as much support and guidance as possible. This will create a favorable environment for innovation among hemodialysis nurses, thereby enhancing their capacity for innovative behavior (Yan et al., 2022).

### 4.2 Correlation analysis of psychological resilience, change fatigue, and innovative behavior among hemodialysis nurses

The Spearman correlation analysis results of this study indicate that psychological resilience among hemodialysis nurses is negatively associated with perceived change fatigue (*r =* −0.467, *p* < 0.001). That is, nurses with higher levels of psychological resilience reported lower perceived change fatigue, consistent with the findings of Gao et al. (2025). Psychological resilience represents an individual’s coping capacity in response to stress and adversity, which may enable rapid recovery or adaptation to environmental changes and may mitigate resource depletion caused by frequent transformations. It may serve as a protective resource against change fatigue, with nurses possessing higher psychological resilience demonstrating greater acceptance of change. Furthermore, a randomized controlled trial by Rogerson et al. (2016) reported that a workplace resilience program improved resilience and reduced perceived stress during a period of significant organizational change; however, its specific effect on change fatigue has not been tested.

The Spearman correlation analysis results of this study indicate that psychological resilience among hemodialysis nurses is positively correlated with innovative behavior (*r =* 0.558, *P* < 0.001). That is, nurses with higher levels of psychological resilience exhibit greater innovative behavior, consistent with the findings of Awad et al. (2025). As a positive psychological state, psychological resilience was positively associated with nurses’ innovative behavior (Lv et al., 2021). Nurses with higher psychological resilience tend to set innovative goals and continuously adapt their methods based on the latest information in the nursing field to achieve these objectives. Nursing managers may consider implementing group coaching and resource support to fully leverage this malleable psychological resource and stimulate innovative behavior, although the effectiveness of such strategies warrants confirmation in longitudinal or experimental studies.

This study’s Spearman correlation analysis revealed a negative correlation between change fatigue and innovative behavior among hemodialysis nurses (*r =* −0.383, *p* < 0.001). That is, lower levels of change fatigue correlate with higher innovative behavior, consistent with findings by Gee et al. (2017). Nurses enduring prolonged change are prone to burnout, intense psychological stress, and professional disengagement, lacking the time, energy, and motivation for innovation (Beaulieu et al., 2023). According to COR theory, managers may continuously replenish nurses’ external resources by fostering a positive organizational atmosphere, providing organizational support, and creating a conducive work environment. This may prevent resource depletion, thereby potentially alleviating psychological fatigue and diminished motivation in response to change, and may be associated with greater innovative behavior (Emiralioğlu and Sönmez, 2021), pending confirmation by future longitudinal or experimental research.

### 4.3 Indirect statistical association of change fatigue between psychological resilience and innovative behavior: an exploratory cross-sectional mediation analysis

The findings reveal that within the exploratory cross-sectional mediation model, change fatigue showed a partial statistical indirect association between psychological resilience and innovative behavior, with an unstandardized indirect association of 0.113, which accounted for 24.4% of the total association; however, this decomposition has limited unstandardized indirect association in cross-sectional data and does not imply a causal mechanism. Specifically, higher levels of psychological resilience among nurses was associated with lower perceived change fatigue and greater willingness to engage in innovative behavior. According to the SOR theory, psychological resilience, as a form of positive psychological capital, may help regulate nurses’ emotions when confronting high-intensity work, unexpected events, or organizational change. This may enable nurses to maintain a sense of meaning and control during change, which may be associated with lower change fatigue. Consequently, nurses may have greater capacity to integrate internal and external resources, enabling them to actively address challenges, propose new methods and technologies, and foster innovative behaviors (Beaulieu et al., 2023). As a mediating factor, lower levels of change fatigue may preserve personal resources, allowing room for trial-and-error, knowledge integration, and autonomous improvement, which may manifest as increased innovative behaviors. Nurses with high psychological resilience may reduce change fatigue through cognitive reappraisal and sense-making, thereby potentially freeing up cognitive resources for innovation. Managers may consider conducting psychological resilience training for nurses while also pacing organizational changes and optimizing communication and resource support to alleviate change fatigue, thus potentially enhancing the innovative potential of hemodialysis nurses, although these implications require confirmation through longitudinal or experimental research.

## 5 Conclusion

This study, grounded in resource conservation theory and the stimulus-organism-response framework, examined the association between psychological resilience and innovative behavior among hemodialysis nurses. Findings indicate that psychological resilience was not only associated with innovation behavior among hemodialysis nurses but also showed a statistical indirect association with innovative behavior through change fatigue. These results provide a theoretical basis for nursing administrators to consider intervention strategies that may enhance innovative behavior in this population. The findings suggest that individual hemodialysis nurses might benefit from psychological resilience training, adopting measures to unlock their inherent potential, fully mobilizing personal initiative, and elevating psychological resilience levels. Hospital administrators might consider fostering supportive environments that leverage organizational, societal, and environmental factors to enhance psychological resilience, potentially establishing comprehensive, multi-perspective psychological resilience enhancement programs. Concurrently, optimizing organizational change models, actively incorporating frontline hemodialysis nurses’ feedback, prioritizing their mental health, and strengthening their coping capabilities may be associated with reduced change fatigue among hemodialysis nurses, which in turn may be linked to greater willingness to engage in proactive innovation, although these implications require confirmation through longitudinal or experimental research.

## 6 Limitations

This study has the following limitations. First, the sample was obtained through convenience sampling from 14 public hospitals in Sichuan Province, China. This non-random sampling method may introduce selection bias, which could limit the generalizability of the findings. Future studies should expand the scope of the survey and conduct a nationwide, multicenter survey with a large sample size. Second, due to limitations in human, material, and financial resources, a cross-sectional survey design was adopted, which does not allow for the determination of temporal sequence. The findings therefore reflect only the current situation and do not permit inferences about causal relationships. Future studies could employ experimental research designs to test causal relationships. Third, factors such as job burnout may also influence innovative behavior. It is recommended that future studies examine alternative mediating variables (e.g., burnout, work engagement, psychological empowerment) and conduct longitudinal studies to explore the association of psychological resilience on the innovative behavior of hemodialysis nurses in Chinese public hospitals. Fourth, although a common latent factor analysis was employed to conduct a statistical assessment of common method bias, since the data were all derived from self-report measurements from a single source, self-report bias and common source bias still cannot be completely eliminated. Moreover, even after adjusting for several key covariates in the mediation model, residual confounding caused by unmeasured variables may still exist. Fifth, given the unique hierarchical culture and nursing workforce allocation in Chinese public hospitals, cultural adaptability must be considered when generalizing these findings to other countries, regions, or non-public hospitals. Finally, because the questionnaires were distributed anonymously through a single online link by department head nurses, hospital-level identifiers were not collected, and we were unable to calculate the intraclass correlation coefficient or apply cluster-robust standard errors directly. While a design-effect sensitivity analysis suggested that our findings were robust to plausible degrees of clustering, the potential non-independence of observations within hospitals cannot be fully excluded. The sample size was estimated based on the precision of the mean score of the primary outcome rather than on a formal power analysis for the mediation model, and the clustered structure of the data was not accounted for in the sample-size calculation, which may have implications for the statistical power of the mediation analysis.

## Data Availability

The original contributions presented in the study are included in the article/supplementary material, further inquiries can be directed to the corresponding author.
